# 

**Published:** 1893-12-16

**Authors:** 


**Notice to Advertisers and Subscribers.**

All Advertisements, Orders for Copies of The Hospital, and Business Communications should be addressed to The Publisher, *not to the Editor*, The Hospital, 428, Strand, W.C.

The Cost of Subscription, post free, is as follows:—Per week, 2½d.; per quarter, 2s. 6d.; per half-year, 5s.; per annum, 8s. 6d Foreign:—Per Annum, 10s. 8d.; payable, in all cases, in advance.

**OUR NEW OFFICES.**

428, Strand, W.C., will henceforth be the Office of this Journal.

**Notices of Births, Deaths, and Marriages** are inserted in this column at a charge of 2s. 6d. for an announcement not exceeding four lines.

**NOTICE TO CORRESPONDENTS.**

All MS., Letters, Books for Review, and other matters intended for the Editor should be addressed The Editor, The Lodge, Porchester Square, London, W.

The Editor cannot undertake to return rejected MS., even when accompanied by stamped directed envelope.

**"Burdett's Hospital Annual," 1893.**

Fourth year of publication. 700 pp. Boards, gilt lettering. A most complete guide to British, Colonial, Indian, and American Hospitals, Dispensaries, Nursing and Convalescent Institutions, and Asylums. Price 5s. Published by The Scientific Press (Limited), 428, Strand, W.C.

**NOTICE.—The Index for Vol. XIV. (ending Sept. 30th, 1893) is on sale at the Office of this Journal, price 2½d., post free.**

### SCRAPS AND GLEANINGS.

A new department for diseases of the skin has just been added to the Sheffield Infirmary.

The Duchess of Portland opened the new Nurses' Residential Home at 17, Nottingham Place, on December 6th.

A cyclists' carnival on an extensive scale took place recently at Southampton to benefit the Free Eye and Ear Hospital.

The Edinburgh charities have profited to the extent of nearly £5,000 by the death of Mr. Charles Jenner, brother of Sir W. Jenner.

The alterations and improvements at the Royal Free Hospital, Gray's Inn Road, will, it is stated, involve an expenditure of £20,000.

The Goldsmiths' Company has given £100 to the Seaside Convalescent Hospital, Seaford, and £500 to the King's College Hospital Special Fund.

The Working Men's Committee of the North Warwickshire Hospital handed over £1,000 to the General Committee of that institution, as the result of their collection efforts.

The Committee of the Boston Hospital in presenting the annual report regret that the balance-sheet shows a deficiency of £181 on this year's accounts, as against £22 in 1892.

The Llanelly Hospital authorities presented a satisfactory financial statement at the annual meeting recently held. The funds of this institution have now £600 at command towards providing increased accommodation.

During the twelve months reviewed in the annual report of the Homœopathic Convalescent Home at Eastbourne 226 persons have been treated in the institution. Subscriptions received during the year amount only to £56.

The Company of Skinners and the Company of Clothworkers have each voted £100 a-year, for the next five years, to King's College Hospital in response to the urgent appeal from that institution for a five years' maintenance fund.

The Derby Children's Hospital at the annual meeting showed a good record of work done during the past twelve months, 1,848 patients being treated during that period. The debt existing on the building fund has now been reduced to £200.

The report of the Medical Board of the North Staffordshire Infirmary stated at the annual meeting that the past year had been marked by an unprecedented increase in the number of patients, 160 more cases having been admitted than in any previous year.

The annual meeting of the West Sussex, East Hants, and Chichester Infirmary and Dispensary was held recently. The finances were reported to be in a satisfactory condition. The private nursing branch has become an increased source of profit to the institution.

A bazaar was held the second week in November at the St. Thomas' Square Chapel, Hackney, in aid of the North-Eastern Hospital for Children. Lady Russell inaugurated and opened this bazaar, with a special view, to aiding an endowment fund for two cots.

The annual bazaar in favour of the funds of the Clevedon Convalescent Home has lost none of its popularity of former years. The last one, which was recently held in the Public Hall, was well attended, and, we learn, proved a substantial help to the above institution.

The Duchess of Teck and the Princess of Wales have sent in articles made by their own hands for disposal at the Children's Salon sale of work, which is to be held on behalf of the cot in the North-Western Hospital on December 20th and 21st at St. Martin's Hall.

### BOOKS RECEIVED.

Williams and Norgate.

"The Supernatural; its Origin, Nature, and Evolution." Two vols. By John H. King.

**Periodicals and Pamphlets.**—The Journal of the Royal Agricultural Society of England, The Young Gentlewoman.

### MEDICAL VACANCIES AND APPOINTMENTS.

### VACANCIES.

The following vacancies are announced this week, the amount of salary in each case being given after the name of the institution:

### Resident Medical Officers.

Eccles and Patricroft Hospital. House Surgeon. Salary £60 per annum, with board and lodging. Applications to the Secretary.

Hospital for Diseases of the Throat, Golden Square, W. Resident Medical Officer. Salary £50 per annum, with board and lodging. Applications to the Secretary by December 18th.

Kimberley Hospital, Kimberley. Senior House Surgeon. Salary £650 per annum, with unfurnished quarters for single men. Applications to H. A. De Beer, Secretary, by December 25th.

Norfolk and Norwich Hospital, Norwich. Assistant to the House Surgeon. Appointment for six months. Board, lodging, and washing provided. Applications to the House Surgeon by December 26th.

North-Eastern Hospital for Children, Hackney Road, N.E. House Surgeon; doubly qualified. Appointment for six months, and at the expiration of that time will be required, if eligible, to serve as Senior House Surgeon for a further period of six months. Salary for the junior post at the rate of £60 per annum, and for the senior post at the rate of £80 per annum. Applications to the Secretary, 27, Clement's Lane, E.C.

Victoria Hospital for Sick Children, Queen's Road, Chelsea, S.W. House Surgeon and House Physician to the In-patients. Honorarium £50 each per annum, with board and lodging in the hospital. Applications to the Secretary by January 13th.

West London Hospital, Hammersmith Road, W. House Physician. Appointment tenable for six months. Board and lodging provided. Applications to R. J. Gilbert, Secretary-Superintendent, by December 27th.

Westminster General Dispensary, Gerrard Street, Soho, W. Resident Medical Officer. Appointment for one year. Applications to the Secretary by December 19th.

### Non-Resident Medical Officers.

City of London Lying-In Hospital, City Road, E.C. District Surgeons. Applications to R. A. Owthwaite, Secretary, by December 19th.

Dental Hospital of London, Leicester Square. Dental Surgeon; must be a Licentiate in Dental Surgery. Applications to J. Francis Pink, Secretary, by January 8th, 1894.

Dental Hospital of London, Leicester Square, W.C. Demonstrator. Honorarium £50 per annum. Applications to Mr. Morton Smale, Dean, by December 18th.

Manchester Royal Infirmary. Junior Administrator of Anæsthetics; non-resident. Salary £50 per annum. Appointment for at least twelve months. Applications to W. L. Saunder, General Superintendent and Secretary.

St. George's Hospital Medical School. Assistant Lecturer in Obstetric Medicine; must be F. or M.R.C.P. Applications to the Dean by December 22nd.

### UNIVERSITIES AND COLLEGES.

### University of Cambridge.

WALSINGHAM MEDAL.—The first award of the High Steward's medal for research in Biology, including Physiology, has been made to Mr. E. W. MacBride, Fellow of St. John's College.

BALFOUR STUDENTSHIP.—This studentship, founded in memory of Professor Frank Balfour, has been awarded to Mr. Arthur Willey, B.Sc. Lond. This is the first occasion on which the Balfour student has not been a member of the university.

MEDICAL EXAMINATIONS.—The plan of the examinations for M.B., B.C., and M.C. has been published in the *University Reporter* for December 5th. The examinations extend from December 8th to the 21st.

PUBLIC HEALTH EXAMINATIONS.—Dr. Ransome, Dr. J. Lane Notter, Dr. Stevenson, and Dr. Thorne Thorne have been appointed Examiners in State Medicine for the current year.

### Conjoint Examinations in Ireland.

At the examinations held by the Conjoint Board in November, 1893, the following gentlemen passed the Final Examination and were granted their Diplomas as Licentiates of the Royal College of Surgeons and the Apothecaries Hall of Ireland: W. A. H. Egerton, E. T. Coady, and J. M Stoker.

### NAVAL AND MILITARY SERVICES.

A garrison class of instruction for Volunteer medical officers is being formed under the direction of the General Officer Commanding the Home District, and will begin the first week in January. There will be about twelve lectures, which will embrace military law, medical organisation, regulations, and official correspondence, &c. The class will be under the direction of the Adjutant of the Volunteer Medical Staff Corps, and officers of the Army Medical Staff, including the following officers: Brigade-Surgeon-Lieutenant-Colonel Evatt, Surgeon-Majors A. E. Hayes and Pope, of Netley, and an officer from Aldershot. Surgeon-Colonel Norton, the Commandant of the London Companies' Volunteer Medical Staff Corps, has lent his headquarters, which are situated in Calthorp Street, Gray's Inn Road, for the use of the class, together with the equipment. All Volunteer medical officers are invited to attend, and those desiring to do so should apply to Surgeon-Captain Hayes, A.M.S., Volunteer Medical Staff Corps, Calthorpe Street.

The following gentlemen have been successful at the recent examination for appointment as surgeon in the Royal Navy: J. P. Willis, M.B., 2,952 marks; W. R. Knightley, 2,692; J. W. W. Stanton, 2,663; A.

Gaskell, 2,622; T. D. Halahan, B.A., M.B., 2,448; J. C. Durston, 2,418; H. N. Stephens, 2,401; E. A. Rogers, 2,398; G. D. B. Levick, 2,317; M. C. Langford, 2,246.

### EDINBURGH TRIPLE QUALIFICATION.

### Papers Recently Set.

#### FIRST EXAMINATION.

Questions in Chemistry. (Three of which are to be answered.)

1. How is Nitric Acid prepared? What are its chief properties? Show by means of equations what occurs when the following nitrates are heated:—(a) Copper Nitrate, (b) Potassium Nitrate, (c) Silver Nitrate, (d) Ammonium Nitrate.
2. What changes occur when aqueous solutions of the following substances are mixed together:—(a) Ammonia and Mercuric Chloride, (b) Caustic Potash and Ammonium Alum, (c) Potassium Iodide and Cupric Sulphate, (d) Nitric Acid and Ferrous Sulphate, (e) Boracic Acid and Sodium Carbonate?
3. How are the following substances prepared:—(a) Chloroform, (b) Iodoform, (c) Chloral?
4. How much Sulphuretted Hydrogen can be got from 100 grams of Ferrous Sulphide? What volume would it occupy at 27° C. and 800 mm. pressure? Fe=56 S=32.

Questions in Elementary Anatomy. (All of which are to be answered.)

1. Describe the Atlas and the Ligaments by which it is connected with the Occipital Bone.
2. Describe the Tibialis Anticus Muscle in regard to its origin, insertion, relations, action, nerve supply, and blood supply.
3. Describe the Median and Radial Nerves in the hand.
4. Name the branches of the Posterior Tibial Artery in their order (except the External Plantar branch).

Questions in Elementary Biology. (Five Questions, of which Four only are to be answered. Illustrate your Answers by means of Sketches.)

1. How do the Protozoa differ from such a form as Hydra?
2. Where is the heart situated in the lobster? Name the principal arteries proceeding from it. What are the alae cordis?
3. The brain of a Frog is exposed by the removal of the fronto-parietal bones and main portion of the roof of the skull. Name the parts of the brain then visible. What are the epiphysis and hypophysis?
4. In a transverse section of the stem of a fern what do you see? Describe a Sporangium.
5. Mention the principal features in the perianth of the Rose. As the Rose does not produce honey, by what means are insects attracted for fertilising purposes, and how are the ripe seeds mainly dispersed?

Questions in Physics. (All of which are to be answered, and calculations shown where required.)

1. Define the resultant of two or more forces. Show how to find the resultant of three forces which act at a point in the same plane.
2. Describe some form of the Force-Pump, and explain its action.
3. Show, by two separate sketches, the action of a double Convex Lens in producing (1) a real image; and (2) a virtual image of a candle-flame.
4. What is the "Thermal Unit"? How many units of heat are required to raise 2 lbs. of iron from 0°C. to 100°C. (the specific heat of iron = 0·12)?

#### SECOND EXAMINATION.

Questions in Advanced Anatomy. (All of which are to be answered.)

1. Describe the dissection to expose the Internal Maxillary Artery in the Pterygo-Maxillary region. Name the branches of the Artery, and give their Anastomoses.
2. Describe the structures which connect the Cerebellum to (a) The Cerebrum, (b) The Pons Varolii, (c) The Medulla Oblongata.
3. What nerves supply the following muscles: (1) Trapezius, (2) Buccinator, (3) Digastric, (4) Crico-thyroid, (5) Rhomboideus Minor, (6) Flexor Profundus Digitorum, (7) Pectineus, (8) Adductor Magnus, (9) Flexor Brevis Pollicis, (10) Flexor Longus Hallucis?
4. Describe the Portal Vein, and give its relations.

Questions in Physiology. (All of which are to be answered.)

1. What are the functions of the Liver? Mention any differences in its function before birth.
2. What changes result from complete division of a Sciatic Nerve?
3. What are the differences between inspired and expired Air?
4. Give an account of the secretion and function of Saliva.

Questions in Materia Medica. (All of which are to be answered).

1. What Oxides of Mercury are official, what are their characters, and what are their doses?

2. Explain the meaning of the term Stearoptene, and give an example.

3. Enumerate the preparations of Red Cinchona Bark, and mention the dose of each.

#### FINAL EXAMINATION.

Questions in Medicine. (Four questions, of which three only are to be answered.)

1. Describe a typical attack of Asiatic Cholera.
2. What symptoms and physical signs would you expect to be present in a case of large Syphilitic Gumma, situated at the base of the Brain, in the region of the Interpeduncular Space.
3. Discuss the pathology and describe the leading symptoms of Addison's Disease.
4. What are the causes of Rickets? Describe a typical case of Rickets.

